# Neonatal Arterial Tortuosity and Adult Aortic Aneurysm—Is There a Missing Link?—A Case Report

**DOI:** 10.3389/fped.2021.814773

**Published:** 2022-03-15

**Authors:** Srabani Bharadwaj, Charmaine Chan, Jonathan Choo Tze Liang, Sarat Kumar Sanamandra, Marielle Valerie Fortier, Ai Ling Koh, Sreekanthan Sundararaghavan

**Affiliations:** ^1^Department of Neonatal and Developmental Medicine, Singapore General Hospital, Singapore, Singapore; ^2^Yong Loo Lin School of Medicine, Singapore, Singapore; ^3^Duke-NUS School of Medicine, Singapore, Singapore; ^4^Lee Kong Chian School of Medicine, Singapore, Singapore; ^5^Cardiology Service, Department of Paediatric Subspecialties, KK Women's and Children's Hospital, Singapore, Singapore; ^6^Department of Radiology, Singapore General Hospital, Singapore, Singapore; ^7^Department of Radiology, KK Women's and Children's Hospital, Singapore, Singapore; ^8^Department of Paediatrics, KK Women's and Children's Hospital, Singapore, Singapore

**Keywords:** neonatal, arterial tortuosity, pediatric, aortic aneurysm, hydrops fetalis

## Abstract

We report a novel case of a full term newborn with non-immune fetal hydrops and arterial tortuosity mimicking a double aortic arch, and cranial fractures in the immediate neonatal period. The infant had no classic features of neonatal arterial tortuosity syndrome or Loeys Dietz syndrome apart from bilateral inguinal hernia. He also had skeletal manifestations in the form of fractures in the neonatal period without any trauma during birth and without clinical evidence of Osteogenesis Imperfecta. A heterozygous missense variant of uncertain significance was detected in *MYH11* gene which is increasingly recognized to be belonging to the familial/hereditary thoracic aneurysm and aortic dissection group of disorders. Fetal hydrops as an association with arterial tortuosity has not been reported in the literature. We hypothesize the possible mechanism behind developing fetal hydrops in this case and discuss the genetic and phenotypic heterogeneity of the Familial Thoracic Aortic Aneurysm and Dissection (FTAAD) group of conditions highlighting the unique phenotypic and genotypic presentations. We recommend a high index of suspicion and vigilance in the early detection of such potentially lethal conditions with sequelae also in adulthood.

## Introduction

Arterial tortuosity, defined as the property of the artery having many turns, is becoming more recognized as a common feature in genetic conditions associated with aortic disease. While arterial tortuosity has been most described in Loeys-Dietz syndrome and Arterial Tortuosity Syndrome (ATS), it has been observed in multiple other genetic disorders associated with aortic dilation and dissection, including Marfan syndrome, aneurysms-osteoarthritis syndrome, and familial thoracic aneurysm and aortic dissection (FTAAD) (1).

Most cases of ATS reported in the neonatal period present with characteristic facial features which include an elongated face, down-slanting palpebral fissures, a beaked nose, a high-arched palate, and micrognathia. Other accompanying manifestations of connective tissue include hyperextensible skin, hernias, and skeletal abnormalities such as arachnodactyly, joint hypermobility, and cutis laxa have also been reported (2–4). In this case report, we describe a term newborn presenting with non-immune fetal hydrops diagnosed with arterial tortuosity, focusing on the possible mechanism of developing hydrops, recognition, management, and recommend a high degree of suspicion in its early detection.

## Case Description

A term male infant with suspected fetal hydrops was delivered by spontaneous vaginal delivery weighing 3,315 gm. The delivery was uncomplicated with no instrumentation. He was intubated and mechanically ventilated at birth. There was no known family history of connective tissue disorders, cataracts or easy bruisability.

Clinical examination confirmed hydrops fetalis with bilateral pleural effusions needing drainage, subcutaneous oedema, and minimal ascites. He was also noted to have multiple fractures involving ribs and left clavicle (**Figure 2**), and a comminuted right parietal skull fracture. There were no dysmorphic features, joint hypermobility, or cutis laxa. His cardiac examination was also normal with no hypertension. He was also noted to have bilateral inguinal hernia once the hydrops resolved.

Echocardiography showed no intracardiac abnormalities but raised suspicion of double aortic arch. The superior “arch” coursed leftward, with the right common carotid and subclavian arteries arising from it, while the hypoplastic arch coursed inferiorly giving rise to the left common carotid and subclavian arteries (**Figures 2E–G**). The maximum velocity across the descending aorta was estimated at 4.59 m/s (pressure gradient 99.9 mmHg). However, the neonate had normal cardiovascular examination with no differential pulse or blood pressure between upper and lower extremities.

**Figure 1:**
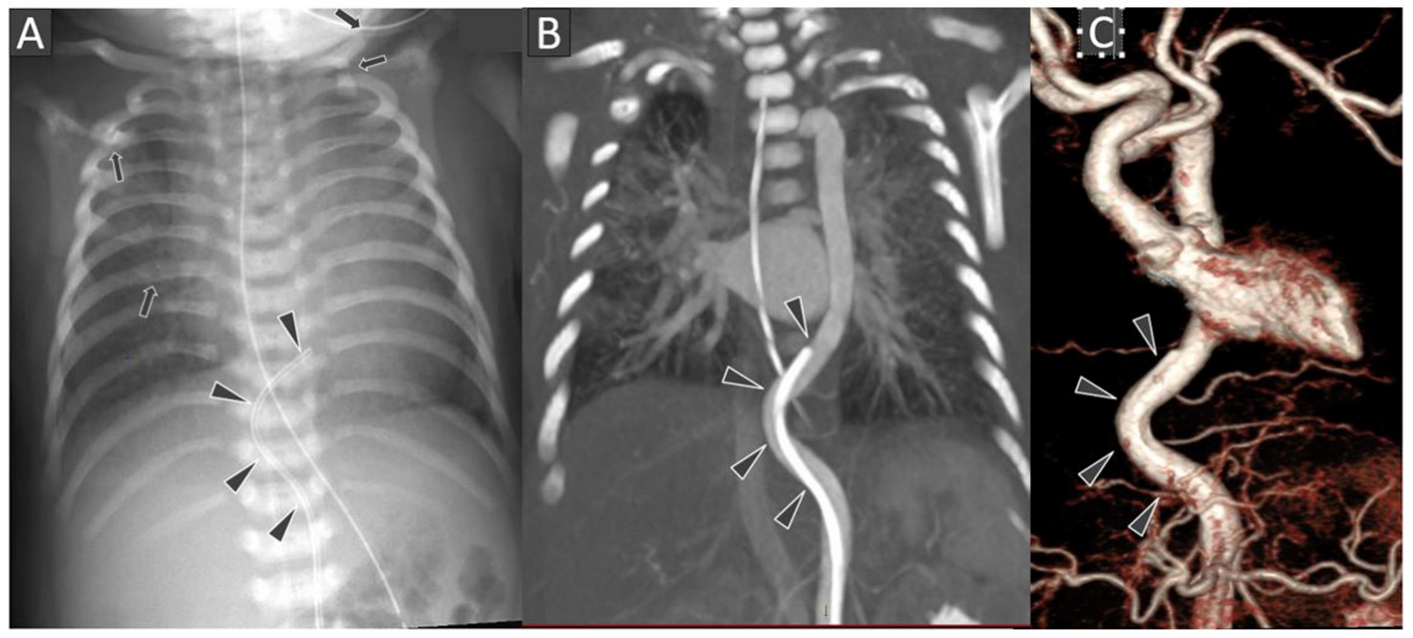
Frontal chest radiograph **(A)** shows the tip of the umbilical arterial catheter (UAC) at the level of T8 vertebral body with curved configuration of its terminal portion (black arrowheads). Several rib and left clavicular fractures (black arrows) are also noted in the bony thoracic cage. Chest CT Angiography coronal multi-planar reconstruction image at the level of thoraco-abdominal aorta **(B)** and 3D surface shaded display image of the thoraco-abdominal aorta **(C)** showing the arterial tortuosity with distal end of the UAC location in descending thoracic aorta.

**Figure 2:**
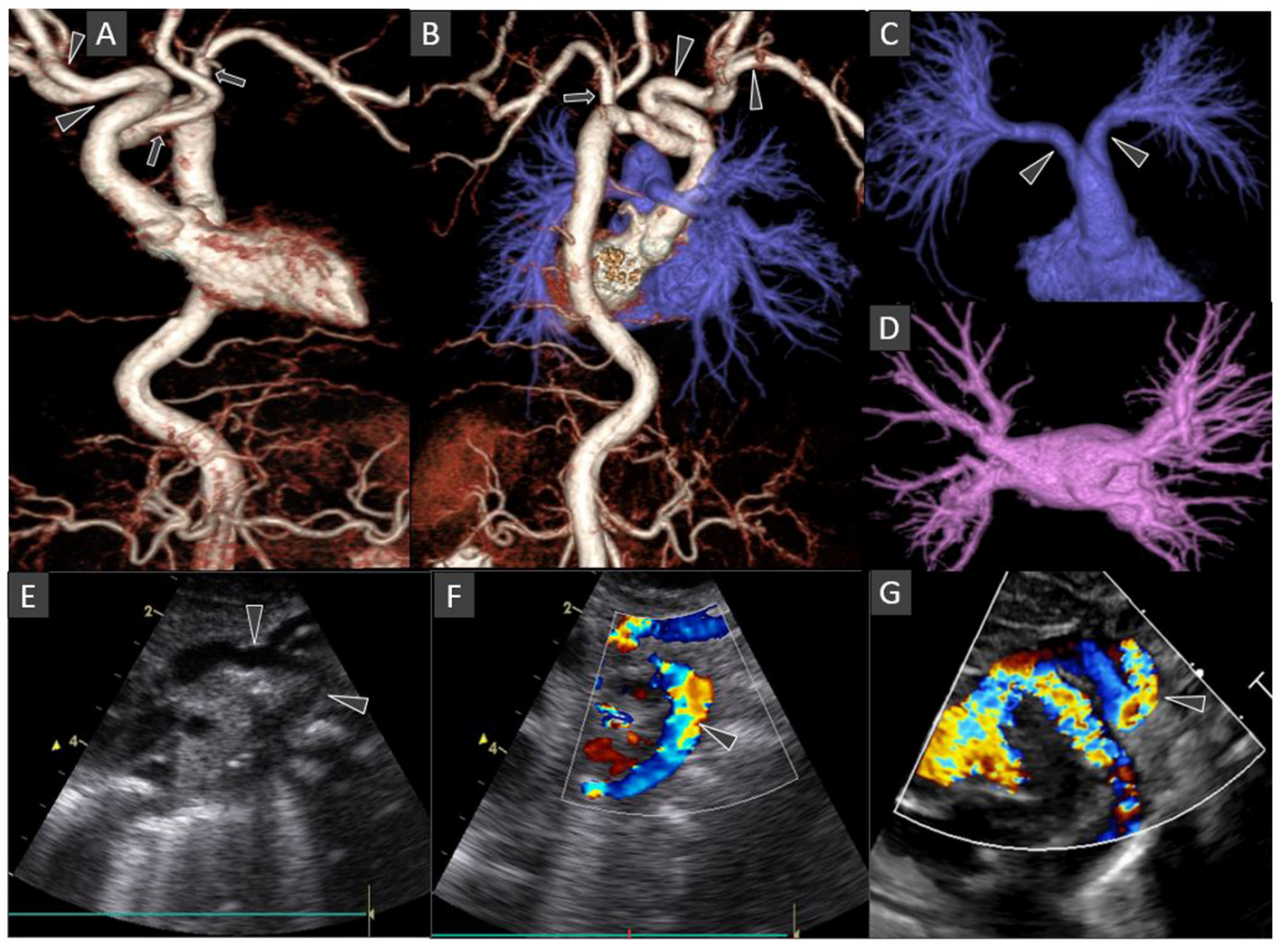
Chest CT Angiography 3D surface shaded display images: Anterior view of the thoraco-abdominal aorta **(A)**; Posterior view of the combined thoraco-abdominal aorta and pulmonary arteries **(B)**; Antero-superior view of the subtracted pulmonary arterial circulation and right atrium **(C)**; Superior view of the subtracted pulmonary venous circulation and left atrium **(D)**. Oblique coronal gray-scale **(E)**; and color Doppler **(F,G)** echocardiography images of the aortic arch and its major branches. The left-sided aortic arch has an unusual configuration with the dilated innominate artery forming a hairpin turn before its bifurcation into right subclavian and common carotid arteries (arrowheads in **A,B**). The left common carotid and subclavian arteries arise from the hypoplastic mid arch (arrows in **A,B**). The thoraco-abdominal aorta is tortuous. Included abdominal aorta shows dilated and tortuous branches of the coeliac axis. The main pulmonary artery is short with early bifurcation with long tubular configuration and parallel proximal course of the right and left main pulmonary arteries (arrowheads in **C**). Normal morphology of bilateral pulmonary veins draining into the left atrium **(D)**. Some of these findings are also demonstrated on echocardiography images (arrows in **E–G**).

The umbilical artery catheter (UAC) (Figure 1) was incidentally noted to have a tortuous course on radiograph on D5 of life (Figure 2A). A Computed Tomography Aortogram (CTA) was performed to evaluate the aortic arch anatomy. CTA confirmed the findings of a left-sided aortic arch with a dilated and tortuous right brachiocephalic artery forming a hairpin loop before bifurcating into the right subclavian and common carotid arteries (Figures 2B,C). The left common carotid and subclavian arteries arose from the hypoplastic transverse arch. The thoraco-abdominal aorta, and branches of the coeliac axis, appeared dilated and tortuous corresponding with the tortuous course of UAC. The branch pulmonary arteries appeared unusual as well, with proximal segments coursing parallel to each other before branching to the left and right, respectively (Figures 2A–C). The pulmonary veins were normal (Figure 2D). The abdominal aorta and the celiac axis branches were dilated and showed tortuosity. The UVC was also tortuous as evidenced in the abdominal roentgenogram.

Genetic tests showed a normal male karyotype and chromosomal microarray analysis. Clinical whole exome sequencing (WES) identified a paternally inherited heterozygous variant of uncertain significance (VUS) in *MYH11* [NM_002474.2(MYH11): c. 1502G>A(p.Arg501His)]. WES also identified a maternally inherited heterozygous likely pathogenic variant in *TRIP4* [NM_016213.4(TRIP4): c.272-1G>T] that is associated with autosomal recessive spinal muscular atrophy with congenital bone fractures 1 (SMABF1) and muscular dystrophy, congenital, Davignon-Chauveau type. However, this result is interpreted as carrier status and less likely to be the cause of multiple birth fractures seen in our patient as he did not exhibit signs of muscle weakness.

All investigations for possible causes of hydrops fetalis were negative including pleural fluid analysis. Patient was investigated for cause of multiple congenital fractures and osteogenesis imperfecta was ruled out with a negative genetic test result. The fractures were managed conservatively and healed completely with no sequelae. The inguinal hernias were repaired surgically. The infant is currently 20 months of age with normal growth and age-appropriate development. He is under regular cardiac and pediatric follow up. The latest echocardiogram shows a moderate gradient across aorta (40 mmHg), no evidence of left ventricular hypertrophy with tortuous descending aorta and pulmonary artery (PA) with mild turbulence noted in branched pulmonary arteries.

## Discussion

Arterial tortuosity syndrome (ATS) is a rare, autosomal recessively inherited connective tissue disorder characterized by severe and widespread tortuosity of the aorta and of middle-sized arteries with an increased risk of aneurysm, dissection, or stenosis involving the aorta, the pulmonary arteries, or both. It is a part of the genetic syndromes with thoracic aortic aneurysm and dissection (TAAD) including Marfan's Syndrome (MS), Loeys Dietz Syndrome (LDS), Ehlers Danlos Syndrome (EDS). Familial TAAD (FTAAD) denotes a group of non-syndromic disorders which generally present with isolated TAAs, without associated characteristic systemic features (5).

Arterial tortuosity is characterized by the presence of abnormal twists and turns of one or several arteries (6). Different types of arterial tortuosity have been described. In 1965, Weibel and Fields referred to S- or C-shaped elongation or undulation of arteries as tortuosity and acute arterial angulation as kinking with its severity ranging from mild (angle ≥60°) to moderate (angle between 30° and 60°) and severe (angle <30°) (7). Barbour et al. described two additional patterns namely looping referring to C- or S-shaped deformity with 2 turns in the vessel with angles <90°, and coiling indicating a 360° turn in the vessel (8).

The exact cause and timing of arterial tortuosity are unclear, although one postulation is that there is abnormal gradual lengthening of the arteries in a fixed space, resulting in forced curving and bending of the vessels (1). The pathophysiological features of ATS include compromised vascular integrity in the arterial vascular beds and disorganized connective tissues overlying blood vessels and even non-vascular structures throughout the body leading to increased flexibility and reduced strength of the vessel wall and other connective tissue (9).

The CTA confirmed all the branches of the aortic arch and pulmonary arteries showing distinctive tortuous course of multiple middle and large sized arteries like the right brachiocephalic artery, the thoraco-abdominal aorta, and coeliac arteries. The arterial tortuosity in this neonate was described as a hairpin loop formed by the dilated and tortuous right brachiocephalic artery before bifurcating into the right subclavian and common carotid arteries and S-shaped tortuosity was noted in thoraco-abdominal aorta and coeliac axis as well. These findings make the index of suspicion for a thoracic artery aneurysm and dissection (TAAD) syndromes very high. Whether it may be a part of FTAAD or syndromic TAAD variants warrants further investigation.

Another unique feature is that the infant presented antenatally with hydrops/hydrothorax. One of the reports detected arterial tortuosity antenatally with abnormally elongated pulmonary artery and a tortuous abdominal aorta, but hydrops was not noted and there was no postnatal genetic confirmation (10). The right brachiocephalic trunk was noted to be similar in size to the left sided hypoplastic aortic arch leading to a suspicion of double aortic arch or a persistent fifth arch on initial echocardiographic assessment. Double aortic arch is reported in the literature to cause fetal hydrops mimicking a congenital high airway obstruction syndrome (CHAOS) which causes pulmonary overdistention due to retained bronchial secretions. Increased intrathoracic pressure with impaired venous return is believed to account for the associated development of fetal hydrops (11). We hypothesize that the bilateral pleural effusions were perhaps secondary to a similar phenomenon with the tortuous arterial course compressing venous/lymphatic drainage (12).

The clinical examination at birth also revealed multiple fractures (depressed comminuted right parietal skull fracture, fracture left clavicle and left first rib) and bilateral inguinal hernia apart from features of hydrops. The fractures were first radiologically documented at day 2 of life when a cranial ultrasound was performed when a 3mm subgaleal hematoma was suspected. Skull roentgenogram done to evaluate this showed a comminuted fracture extending to the occipital bone. There was also a right parieto-temporal swelling noted since birth. Clinically there were no other features suggestive of OI, skeletal dysplasia/ bony deformities. No family history of OI as well. However, the infant did not develop any new fractures so far. There were no features of cutis laxa, facial dysmorphism or hypermobile joints/dislocations, keratoconus which is often described in ATS. The absence of any associated dysmorphism makes the suspicion of a FTAAD higher.

Coarctation of aorta and severe hypertension in infancy have both been reported in neonates with arterial tortuosity (13, 14). The pressure gradient across the descending aorta was estimated at 99 mmHg, although the doppler tracing did not show any diastolic tail, indicating “pseudo-coarctation” from kinking. The infant did not present with any neonatal hypertension and neither was there any discernable blood pressure difference between the upper and lower extremities. Whether the pseudo-coarctation, would manifest later remains to be seen.

Clinical WES did not identify any pathogenic variant in *SLC2A10* gene that is associated with ATS. A paternally inherited heterozygous missense VUS c.1502G>A(p.Arg501His) was identified in *MYH11* which is not specifically described in ATS but reported in FTAAD and as part of generalized aortopathies (15–17). This missense variant c.1502G>A(p.Arg501His) has been previously reported in one individual with abdominal aortic aneurysm (18), one individual with thoracic aortic aneurysm and dissection who also had another variant of uncertain significance in *FBN1* c.8069T>G(p.Met2690Arg) (19) and one individual with spontaneous coronary artery dissection in coexistence with another variant of uncertain significance in *FBN2* c.4141C>A(p.His1381Asn) (20). None of these individuals was reported to have ATS. This variant has been observed at a frequency of 0.02% (63/282,888 alleles) with the highest allele frequency, observed being 0.11% in the East Asian population (gnom AD) (21). The arginine codon 501 is highly conserved and computational analyses (SIFT, Poly Phen-2) predict that this variant is deleterious (22, 23). However, the variant is classified as uncertain significance due to insufficient information to support pathogenicity. Our patient's father was not known to have any significant cardiovascular condition. In view of the potential risk of aortic aneurysm and dissection, cardiovascular screening for aortic aneurysm was recommended for the father.

Recent reports have identified a heterogenous phenotype and identified many new mutations as part of hereditary thoracic aortic aneurysm and dissection (HTAAD) aortopathies (17, 21). ATS has been associated with mutations in the *SLC2A10* gene, but *MYH11* has been increasingly reported in FTAAD (16–18, 21). Loup et al. reported a case of arterial tortuosity with coarctation of aorta noted as an incidental finding in a 14 month old baby with no significant family history or positive genetic test result for commonest ATS mutations but targeted sequencing of *MYH11* identified a maternally inherited heterozygous missense variant in *MYH11* c.2075C>T in exon 17 (13), which was different from the variant detected in our patient. *MYH11* is classified to be one of the genes which are definitively associated with HTAAD (24). However, the genotype-phenotype association between *MYH11* and ATS remains uncertain.

The limitations of the case report are in the difficulty in not having a genetic confirmation of the phenotype, but it is well recognized now that FTAAD is a spectrum and not all cases of FTAAD have reported genetic mutations. The novelty of the case is in its prompt detection in the neonatal period which will aid in keeping the infant and under surveillance and monitor progression as later development of pulmonary stenosis and coarctation of aorta has been reported in the literature (25).

## Conclusion

Any patient with suspected aortic arch abnormality and tortuosity should be evaluated for both anatomic and functional changes. Our case highlights the phenotypic and genotypic heterogeneity of TAAD with unique antenatal and neonatal presentation. This also shows the distinctive but potential association between arterial tortuosity and late onset adult phenotypes such as TAAD. Having a high index of suspicion in patients with suspected tortuous vascular anatomy will aid in early pick up of syndromic and non-syndromic conditions with potentially devastating sequelae in adulthood.

## Data Availability Statement

The original contributions presented in the study are included in the article/supplementary materials, further inquiries can be directed to the corresponding author.

## Ethics Statement

Ethical review and approval was not required for the study on human participants in accordance with the local legislation and institutional requirements. Written informed consent to participate in this study was provided by the participants' legal guardian/next of kin.

## Author Contributions

SB wrote the initial manuscript and led revisions. CC contributed to literature review and reviewed the manuscript. JC contributed in the cardiological findings including echocardiographic correlation and critically reviewed and revised the manuscript. SS conceived the report, clinical evaluation, echocardiographic correlation, and critical review and revision of the manuscript. SKS and MF contributed to the images and its clinical correlation and critically reviewed the manuscript. AK provided the genetic input and critically reviewed and revised the manuscript. All authors approved the final manuscript as submitted and agree to be accountable for all aspects of the work.

## Conflict of Interest

The authors declare that the research was conducted in the absence of any commercial or financial relationships that could be construed as a potential conflict of interest.

## Publisher's Note

All claims expressed in this article are solely those of the authors and do not necessarily represent those of their affiliated organizations, or those of the publisher, the editors and the reviewers. Any product that may be evaluated in this article, or claim that may be made by its manufacturer, is not guaranteed or endorsed by the publisher.

## Abbreviations

UAC: umbilical artery catheter
UVC: Umbilical venous catheter
ATS: Arterial Tortuosity Syndrome
FTAAD: Familial Thoracic Aortic Aneurysm and Dissection.
